# The SCREENIVF Hungarian version is a valid and reliable measure accurately predicting possible depression in female infertility patients

**DOI:** 10.1038/s41598-024-63673-w

**Published:** 2024-06-05

**Authors:** Judit Szigeti F., Réka E. Sexty, Georgina Szabó, Csaba Kazinczi, Zsuzsanna Kéki, Miklós Sipos, Péter Przemyslaw Ujma, György Purebl

**Affiliations:** 1 Institute of Behavioral Sciences, Semmelweis University, Üllői út 26, 1085 Budapest, Hungary; 2 Department of Otorhinolaryngology, Head and Neck Surgery, Semmelweis University, Üllői út 26, 1085 Budapest, Hungary; 3 Department of Psychology, University of Graz, Dachgeschoß – 2, Stock, 2, 8010 Graz, Austria; 4 Doctoral School of Mental Health Sciences, Semmelweis University, Üllői út 26, 1085 Budapest, Hungary; 5 Department of Psychiatry, North Buda Saint John’s Hospital Center and Outpatient Clinic, Diós Árok 1-3, 1125 Budapest, Hungary; 6 Department of Clinical Psychology, Semmelweis University, Budapest, Hungary; 7 Doctoral School of Clinical Medicine, University of Szeged, 6722 Szeged, Hungary; 8 Directorate for Human Reproduction, National Directorate General for Hospitals, Buda-part tér 2, BudaPart Gate Irodaház A. ép. 406, 1117 Budapest, Hungary; 9 Department of Obstetrics and Gynecology, Assisted Reproduction Center, Semmelweis University, Üllői út 26, 1085 Budapest, Hungary

**Keywords:** Psychology, Health care

## Abstract

Infertility patients, often in high distress, are entitled to being informed about their mental status compared to normative data. The objective of this study was to revalidate and test the accuracy of the SCREENIVF, a self-reported tool for screening psychological maladjustment in the assisted reproduction context. A cross-sectional, questionnaire-based online survey was carried out between December 2019 and February 2023 in a consecutive sample of female patients (N = 645, response rate 22.9%) in a university-based assisted reproduction center in Hungary. Confirmatory factor analysis and cluster and ROC analyses were applied to test validity, sensitivity and specificity in relation to Beck Depression Inventory (BDI) scores. Model fit was optimal (chi-square = 630.866, p < 0.001; comparative fit index = 0.99; root-mean-square error of approximation = 0.018 (90% CI 0.013–0.023); standardized-root-mean-square-residual = 0.044), and all dimensions were reliable (α > 0.80). A specific combination of cutoffs correctly predicted 87.4% of BDI-scores possibly indicative of moderate-to-severe depression (χ^2^(1) = 220.608, p < 0.001, Nagelkerke R^2^ = 0.462, J = 66.4). The Hungarian version of the SCREENIVF is a valid and reliable tool, with high accuracy in predicting BDI-scores. Low response rate may affect generalizability. The same instrument with different cutoffs can serve various clinical goals.

## Introduction

Infertility is a chronic disease commonly accompanied by psychological symptomatology^1^. The rate of infertile women who meet the criteria for a psychiatric diagnosis may reach 40%^2^, with depressive^3,4^ and anxiety disorders^5^ being the most prevalent within clinical samples. Women are almost unanimously found to present higher levels of mental health problems connected to infertility than men^6^. If distress reaches mental disorder levels, it also seems to hinder the success of assisted reproduction techniques (ART)^7^.

Psychosocial interventions increase psychological well-being and the likelihood of pregnancy in infertility patients^8–11^. However, there is a large disparity between the number of patients reporting infertility distress and the number of those seeking psychological support^12^. Furthermore, patients’ mental health status deteriorates with unsuccessful attempts. Therefore, it is necessary that patients (1) be informed about their mental status compared to normative data and (2) should be checked at various time points during infertility treatment with properly validated mental health instruments.

Several psychometric tools exist to assess adjustment to infertility, the methodological trend moving from the use of generic tools, also applicable to infertile patients, to instruments developed specifically for this population. The European Society of Human Reproduction and Embryology guideline^13^ lists 12 such tools, 5 generic and 7 specific. Out of the ones assessing mental health and not, say, perceived quality of care, experience of patient-centeredness, or fertility status awareness, the most comprehensive and widely used instrument is the FertiQoL^14,15^, assessing patient needs in three domains: behavioral, emotional, and social-relational. The one recommended for screening purposes is the SCREENIVF^16^, a combination of both generic and specific items, also assessing three domains (emotional, social-relational and cognitive), and quick to complete. Out of the existing fertility-related questionnaires, only the FertiQoL and the SCREENIVF have been validated in Hungarian^17,18^.

The SCREENIVF consists of five subscales measuring anxiety, depression, social support, cognitions of helplessness and lack of infertility acceptance, each based on a reflective model, that is, one in which all items are a manifestation of the same underlying construct^19^. The tool has been validated in Portuguese^20^, Dutch^21^, and Turkish^22^. Cutoff values are not uniform in these studies, and there are considerable deviations in the rates of patients found to be at risk. A Hungarian validation study was performed on a small sample (N = 60), showing good reliability and model fit but low specificity^18^. Therefore, further validation of the Hungarian version seems justified.

The aim of the present study was to reinvestigate the psychometric characteristics and screening capacities of the Hungarian SCREENIVF on a larger sample of Hungarian women in assisted reproduction. We hypothesized that (1) the psychometric properties of the SCREENIVF can remain convincing and (2) the SCREENIVF is able to screen out ART patients to be further explored for mood disturbances if cutoffs for the Depression subscale and the SCREENIVF Risk Factor scale are tested against real data and applied accordingly.

## Results

Study participants were in their mid-30s (Table 1), almost all in legally sanctioned heterosexual relationships, qualified, employed and financially secure. The mean duration of, mainly primary, infertility was 3.3 years. Etiology was varied, with one-third unexplained or (yet) unknown. The existence of other chronic diseases was not typical, but being overweight was rather frequent.

**Table 1:** Sociodemographic and medical characteristics of the sample.

Sociodemographic data		
Age (M^a^ ± SD^b^)	35.72	4.62

	N^c^	%^d^
Marital status		
Married	503	77.98
Cohabiting	134	20.78
Single	8	1.24
Number of existing children		
0	594	92.10
1	44	6.82
2	7	1.08
Residence		
Capital	364	56.43
City	43	6.67
Town	164	25.43
Village	71	11.01
Other	3	0.46
Education		
Primary	4	0.62
Secondary vocational	9	1.40
Secondary GCE	58	8.99
Postgraduate vocational	64	9.92
College/university	468	72.56
Doctoral studies	35	5.43
Other	7	1.08
Employment status/position		
Employed, subordinate	459	71.16
Employed, mid-level	82	12.71
Executive/Manager	30	4.65
Self-employed	53	8.22
Unemployed	2	0.32
Other	19	2.94
Personal perception of financial situation (M ± SD) (from 1 = *very bad* to 10 = *very good* in relation to average)	6.44	1.51
Health information		
Body mass index (M ± SD)	23.77	4.81
Underweight: < 18.50	34	5.30
Normal weight: 18.50–24.99	407	63.10
Overweight: 25.00–29.90	143	22.20
Obese: > 29.90	59	9.10
Outlier	2	0.30
Duration of infertility (in months) (M ± SD)	40.22	29.51
Etiology of infertility		
Female	222	34.42
Male	62	9.61
Combined	149	23.10
Unexplained	132	20.47
Doesn’t know/no work-up yet	80	12.40
Other chronic disease		
Yes	94	14.57
No	551	85.43
Total	645	100

^a^Mean.

^b^Standard deviation.

^c^Number.

^d^Percentage.

### Reliability and validity

Reliability tests resulted in good to excellent Cronbach’s alpha values (Table 2).

**Table 2:** Descriptive statistics of SCREENIVF subscales with Cronbach’s alpha values.

SCREENIVF subscales	Number of items	Cronbach’s alpha	M^a^ ± SD^b^	Range
Anxiety	10	0.889	22.54 ± 6.87	10–40
Depression	7	0.810	3.54 ± 3.16	0–21
Social support	5	0.901	17.72 ± 3.09	5–20
Helplessness	6	0.867	13.76 ± 4.70	6–24
Acceptance	6	0.928	12.48 ± 4.55	6–24

^a^Mean.

^b^Standard deviation.

All item-subscale correlations were higher than 0.40, indicating that the items contribute sufficiently to their subscales, except for one depression item, referring to suicidal ideation (item 7, 0.381; Supplementary Table S1). Substantial correlations were found between the SCREENIVF subscales (Table 3), with the highest between Depression and Anxiety, and the lowest between Helplessness and Social Support.

**Table 3:** Spearman’s rank correlation coefficients between SCREENIVF subscales, risk factors and cognate measures.

SCREENIVF	Anxiety	Depression	Social support	Helplessness	Acceptance	Risk factors
Anxiety	1					
Depression	0.812**	1				
Social support	− 0.470**	− 0.486**	1			
Helplessness	0.589**	0.638**	− 0.336**	1		
Acceptance	− 0.586**	− 0.597**	0.405**	− 0.627**	1	
Risk factors	0.704**	0.726**	0.545**	0.641**	− 0.595**	1
STAI-S^a,b^	0.769**	0.719**	− 0.441**	0.621**	− 0.620**	0.654**
STAI-T^a,c^	0.755**	0.739**	− 0.510**	0.623**	− 0.615**	0.683**
BDI^a,d^	0.800**	0.908**	− 0.514**	0.627**	− 0.581**	0.704**
MSPSS^e^	− 0.475**	− 0.485**	0.600**	− 0.391**	0.429**	− 0.505**
FertiQoL^f^ emotional	− 0.626**	− 0.653**	0.377**	− 0.764*	0.712**	− 0.602**
FertiQol mind–body	− 0.664**	− 0.706**	0.421**	− 0.749**	0.612**	− 0.630**
FertiQol relational	− 0.412**	− 0.358**	0.493**	− 0.292**	0.331**	− 0.410**
FertiQol social	− 0.549**	− 0.592**	0.436**	− 0.675**	0.571**	− 0.572**
Core FertiQol total	− 0.716**	− 0.737**	0.542**	− 0.791**	0.702**	− 0.717**

Bold letters indicate high (> 0.7) to very high (> 0.9), and italics are used for moderate (0.5–0.7) correlations.

^a^Except for overlapping items.

^b,c^Spielberger State-Trait Anxiety Inventory – State/Trait subscale.

^d^Beck Depression Inventory.

^e^Multidimensional Scale of Perceived Social Support.

^f^Fertility Quality of Life Scale.

**Significant at the 0.01 level.

**Figure 1:**
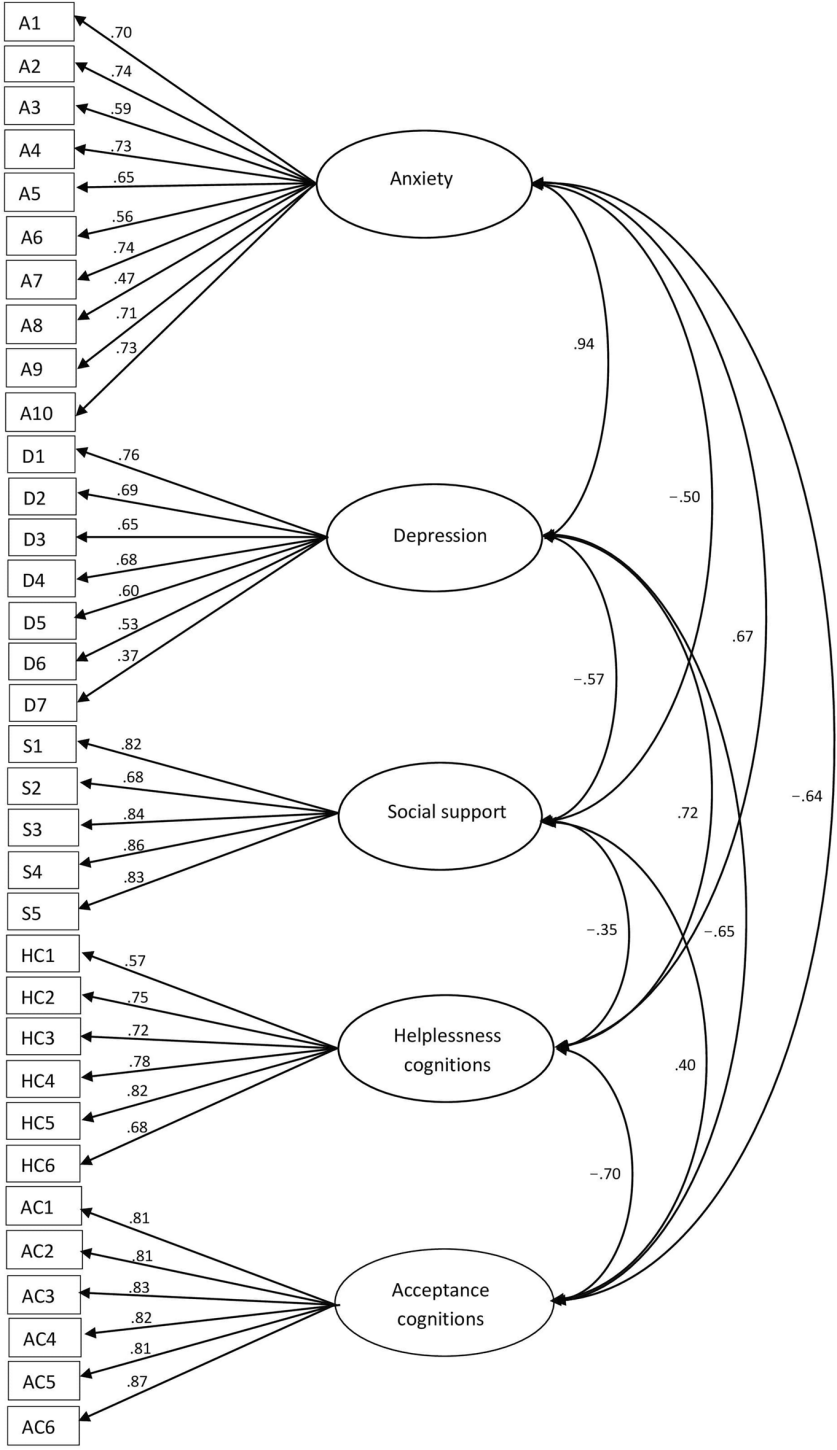
Standardized regression weights of factor loading. *A* anxiety subscale item, *D* depression subscale item, *S* social support subscale item, *HC* helplessness cognitions subscale item, *AC* acceptance cognitions subscale item.

CFA with DWLS indicated optimal model fit (χ^2^ = 630.866, p < 0.001, RMSEA = 0.018 [CI90 = 0.013–0.023], CFI = 0.998, TLI = 0.997, SRMR = 0.042). Standardized factor loadings ranged from 0.4 to 0.9, confirm to the majority of empirical research studies^23^, except for, again, the Depression item on suicidal ideation, which had a weak factor loading (0.37; Fig. 1). CFA without the suicide-related Depression item, however, showed worse model fit than with it (χ^2^ = 615.692, p < 0.001, RMSEA = 0.020 [CI90 = 0.015–0.025], CFI = 0.997, TLI = 0.997, SRMR = 0.043).

As hypothesized, all correlations between SCREENIVF subscales and cognate measures were significant, and most of them were fairly strong (Table 3), the highest when the subscale and the external tool measured the same construct, e.g. Depression and the BDI, Anxiety and the STAI-S, Social Support and the MSPSS. The Helplessness subscale related best to the Core FertiQoL, and the Acceptance subscale with FertiQol Emotional. The SCREENIVF Risk Factors total score showed the strongest relationship with the Core FertiQoL, but was also related well to the BDI.

### Cutoffs, sensitivity and specificity

Based on BDI results, 50.1% (N = 323) of our population was unaffected by depressive symptoms, 30.4% (N = 196) displayed mild symptoms, and 19.5% (N = 126) displayed moderate to severe, that is, clinically relevant symptomatology. The various cutoff values and corresponding ‘at risk’ sample rates in the original study, the five validation studies and the present study are listed in Table 4. All overall models of SCREENIVF risk categories (‘at risk’ vs ‘not at risk’) as predictors of BDI categories (‘case’ vs ‘no-case’ and ‘no-to-mild’ vs ‘moderate-to-severe depression’) were statistically significant when compared to the null model, irrespective of Depression subscale cutoff point.

**Table 4:** Cutoffs and rates of female population found to be ’at risk’ in SCREENIVF validation studies.

	Anxiety	Depression	Social support	Helplessness	Acceptance	Risk factors scale
Verhaak et al. (2010)						
Cutoff	≥ 24	≥ 4	≤ 15	≥ 14	≤ 11	≥ 1
At risk	10.0%	11.0%	16.0%	16.0%	16.0%	34.0%
Lopes et al. (2014)						
Cutoff	≥ 27	≥ 4	≤ 15	≥ 15	≤ 11	≥ 1
At risk	18.4%^a^	28.1%^a^	18.3%^a^	21.7%^a^	18.3%^a^	52.2%^b^
Ockhuijsen et al. (2017)						
Cutoff	≥ 24	≥ 4	≤ 15	≥ 14	≤ 11	≥ 1
At risk	25.3%	21.5%	23.3%	30.9%	18.2%	52.0%
Irmak Vural et al. (2021)						
Cutoff	≥ 24	≥ 2	≤ 15	≥ 14	≤ 7	≥ 1
At risk	16.0%	16.0%	2.0%	8.2%	13.9%	No data
Prémusz et al. (2022)						
Cutoff	≥ 24	≥ 4	≤ 15	≥ 14	≤ 11	≥ 1/ ≥ 2
At risk	24.1%	56.9%	37.8%	25.9%	20.7%	> 90%/50%
Present study						
Cutoff	≥ 30	≥ 4^c^	≤ 14	≥ 19	≤ 7	≥ 1/≥ 2
At risk	18.6%	44.2%	13.6%	17.5%	14.4%	50.9%/31.9%
Cutoff		≥ 5^d^				≥ 1/≥ 2
At risk		34.3%				45.0%/29.3%
Cutoff		≥ 7				≥ 1/≥ 2
At risk		17.8%				39.2%/22.8%

For depression, Verhaak et al., Lopes et al. and Ockhuijsen et al. based their cutoffs on Beck et al. Prémusz et al. took over all cutoffs from Verhaak et al. All other cutoffs were determined by mean plus/minus standard deviation values of actual samples, if not indicated otherwise.

^a^Men included.

^b^Data exclusively on women.

^c^Based on Beck et al.^24^.

^d^Based on cluster analysis.

The best equation results were found in two cases: first, when a 3/4 cutoff on the SCREENIVF Depression subscale and a 0/1 cutoff on the SCREENIVF Risk Factor scale were applied, a procedure correctly predicting 84.5% of BDI depression cases vs no-cases (χ^2^(1) = 220.246, p < 0.001, Nagelkerke R^2^ = 0.386, Youden index corresponding to the cutoff: J = 69.0). Second, when a 6/7 cutoff on the SCREENIVF Depression subscale and a 1/2 cutoff on the SCREENIVF Risk Factors scale were applied, a procedure correctly predicting 87.4% of BDI moderate-to-severe vs no-to-mild depression cases (χ^2^(1) = 220.608, p < 0.001, Nagelkerke R^2^ = 0.462, Youden index corresponding to the cutoff: J = 66.4; Supplementary Table S2).

Two-step cluster analysis of the SCREENIVF Risk Factors scale confirmed a natural demarcation between scores 1 and 2 (Supplementary Fig. S1). ROC analysis showed the best result when the SCREENIVF Risk Factors scale was used with a 4/5 cutoff on the Depression subscale, with BDI no-to-mild vs moderate-to-severe depression applied as a state variable (AUC = 0.918, CI [0.895, 0.940]). ROC curves indicated that the SCREENIVF had a better diagnostic value when used to discriminate between no-to-mild and moderate-to-severe BDI cases of depression (AUCs between 0.647 and 0.898) than when distinguishing cases from no-cases (AUCs between 0.603 and 0.795). When ROC curves for all individual and total SCREENIVF risk factors were compared, the AUC for the total factor (0.898) was superior to that of all subfactors, including Depression (0.848; Supplementary Fig. S2).

## Discussion

The aim of this study was to revalidate the Hungarian version of the SCREENIVF on a fairly large sample and to test its ability to grasp emotional maladjustment with various cutoffs on the Depression subscale and the total Risk Factors scale. The SCREENIVF proved to be a valid and reliable instrument to detect emotional maladjustment among Hungarian women undergoing ART, and was able to discriminate between scores possibly indicative of different levels of depression as measured by the BDI.

Our respondents were typical of ART patients in terms of help-seeking age (mid-30s) in developed countries^25^, where the age of family formation is constantly increasing^26^. Additionally, our sample displayed fairly high socioeconomic status, a common finding among patients in ART^27^. The homogeneity of our sample’s marital status reflects Hungarian legislation, namely, that ART is allowed only for married or officially cohabitating opposite-sex couples and single women who cannot have children otherwise.

The unfortunate coincidence of the study timelines with the COVID-19 pandemic posed considerable challenges. Participant enrolment practically stopped during the first wave, when only life-saving operations were permitted. As an exception, however, immediately after the first wave, the Hungarian health regulations allowed for assisted reproductive interventions to be performed. Even so, with the threat of treatment cancellations due to patients’ virus infection, partners not being allowed to accompany women to examinations and interventions, and the general existential and financial concerns, COVID-19 most probably influenced the stress of infertility^28^. Unfortunately, our original, preregistered study protocol did not allow us to investigate the added effect of the pandemic on the mental wellbeing of the participants.

Online data collection may have introduced sampling bias due to low response rate, possibly caused by overlooking the announcement, interpreting it as junk mail, or completion interrupted by technical or personal difficulties^29^. Our response rate (22.9%) was lower than average proportions found in meta-analyses (34–36%)^30,31^. This was not attributable to a lack of internet access, since we were able, with permission, to extract from the medical database email addresses for all patients, who thus had equal opportunities to fill in the questionnaire, with illegitimate participation also minimized. Low response rates do not necessarily indicate large nonresponse error, i.e. that nonrespondents would have provided different answers than actual respondents^32^.

All of our Cronbach’s alpha values fall within the intervals found in previous studies. The Depression subscale also fits in the tendency of being the least consistent, with the suicidality item not contributing well to its reliability. Even so, the subscale proved more reliable than in Irmak Vural and colleagues’ study^22^, where four items had to be left out. Despite the poor factor loading of the suicidality item, we do not recommend its removal, since (1) model fit indices did not improve with its removal, and (2) the item may warn of suicide risk, signaling an urgent need for further exploration. This is of utmost importance in Hungary, which is customarily among the first five highest suicide-rate states in Europe, e.g. second highest in 2020 (https://ec.europa.eu/eurostat/web/products-eurostat-news/w/edn-20230908-3).

Although two of the previous validation studies of the SCREENIVF^20,21^ used parcel- rather than item-based CFA, here, DWLS estimation yielded excellent results. DWLS is specifically designed for categorical (e.g., binary or ordinal) data in which neither the normality assumption nor the continuity property is plausible^23^, especially for higher sample sizes^33^. Therefore, the hypothesis of the five latent variables underlying the SCREENIVF seems to be confirmed.

Previous literature on the SCREENIVF draws attention to probable transcultural differences in cutoffs^20^ and to a need for ROC analyses in establishing them^21^. We decided to test cutoff points in relation to a ‘gold standard’ measure, the BDI, because depression tools are commonly used as criterion measures of psychological maladjustment in infertility^34^. We found that the SCREENIVF is able to detect possible depression, reaching the best sensitivity with a 3/4 cutoff on the Depression subscale and 0/1 on the Risk Factors scale. Additionally, it is able to identify presumable moderate-to-severe cases of depression, showing the best specificity with a 6/7 cutoff on the Depression subscale and 1/2 on the Risk Factors scale. When the goal is a good trade-off between sensitivity and specificity, the SCREENIVF Risk Factors scale is best used with a 4/5 cutoff on the Depression subscale, to separate no-to-mild cases from those to be further evaluated for moderate-to-severe depression.

Therefore, if one wants to use the SCREENIVF to identify the highest number of possible cases of psychological disorders, a lower cutoff value is needed to maximize sensitivity. If, however, the SCREENIVF is intended to find individuals most likely already affected by depression, a higher cutoff value is needed to maximize specificity. Our analysis showed that the SCREENIVF serves both goals fairly well as a stand-alone tool able to signal different levels of emotional maladjustment, depending on the purposes of the clinician.

Ockhuijsen et al.^21^ found that the SCREENIVF did not do well at prognosticating whether ‘at risk’ patients would actually develop psychological problems during treatment. We found that the SCREENIVF has high screening accuracy at the time of administration. Thus, we believe that the SCREENIVF can safely be used for screening out ‘afflicted’ rather than ‘at-risk-for-later-maladjustment’ patients, who can then be referred to a full diagnostic evaluation. Additionally, given that the ROC curve for the SCREENIVF Risk Factors total scale outperformed that of the Depression subscale, it seems that there is more to the emotional disturbance in infertility than just depressed mood: anxiety, helplessness, lack of social support and unacceptance of infertility all add their own shades to the clinical aspect.

ROC analysis can be combined with utility-based decisions to determine optimal cutoff points^35^, in this case, a desirable balance between the number of patients found vulnerable and the financial costs of delivering psychosocial support. In Hungary, where there is a scarcity of mental health professionals in state-subsidized facilities, it is essential that we single out patients most in need of psychological services by applying higher thresholds on SCREENIVF scales. The proportion (22.8%) of the possibly afflicted population spotted in this sample is realistic in terms of healthcare possibilities on the Eastern European scene, while also comparable to international prevalence data.

A great strength of our study is the use of four cognate measures, all validated in Hungarian, to test the convergent validity of the SCREENIVF, two of them measuring general psychological distress, and two assessing infertility-related issues. Additionally, arbitrariness was ruled out by relying on statistical methods that reveal natural groupings in real-life data. We worked with a relatively sizable sample from a large fertility center in the capital that also serves IVF patients from the rest of the country.

The study has some limitations. Including only women made it impossible to validate the SCREENIVF in men or at the couple level. The cross-sectional design did not allow for testing the predictive validity of the SCREENIVF. There was only one measurement point, so neither test–retest reliability nor responsiveness was assessed. We did not collect data on ART treatment type, so we could not test either the tool’s discriminative validity or the possibly changing status of the patients in different stages of ART. Form X of the STAI was used here, a version still predominantly administered in Hungary, a circumstance that, however, may make international comparisons problematic. Online sampling resulted in a lower than average response rate, which may decrease generalizability. Finally, the overlap of our study timelines with the COVID-19 pandemic may be a source of bias, since our design made it impossible to discern infertility stress per se from the distress caused by COVID-specific effects. Future studies are warranted to overcome the above shortcomings.

## Conclusions

Our findings demonstrated the SCREENIVF to be a valid and reliable measure with high accuracy in predicting possible depression, which gives it significant clinical value in assessing the psychological status of Hungarian women pursuing ART. Given its fair screening precision and goal-adjusted flexibility, we highly recommend its administration in routine fertility care to promote mental health and thus raise the probability of treatment adherence and success.

## Methods

### Study participants

**Figure 2:**
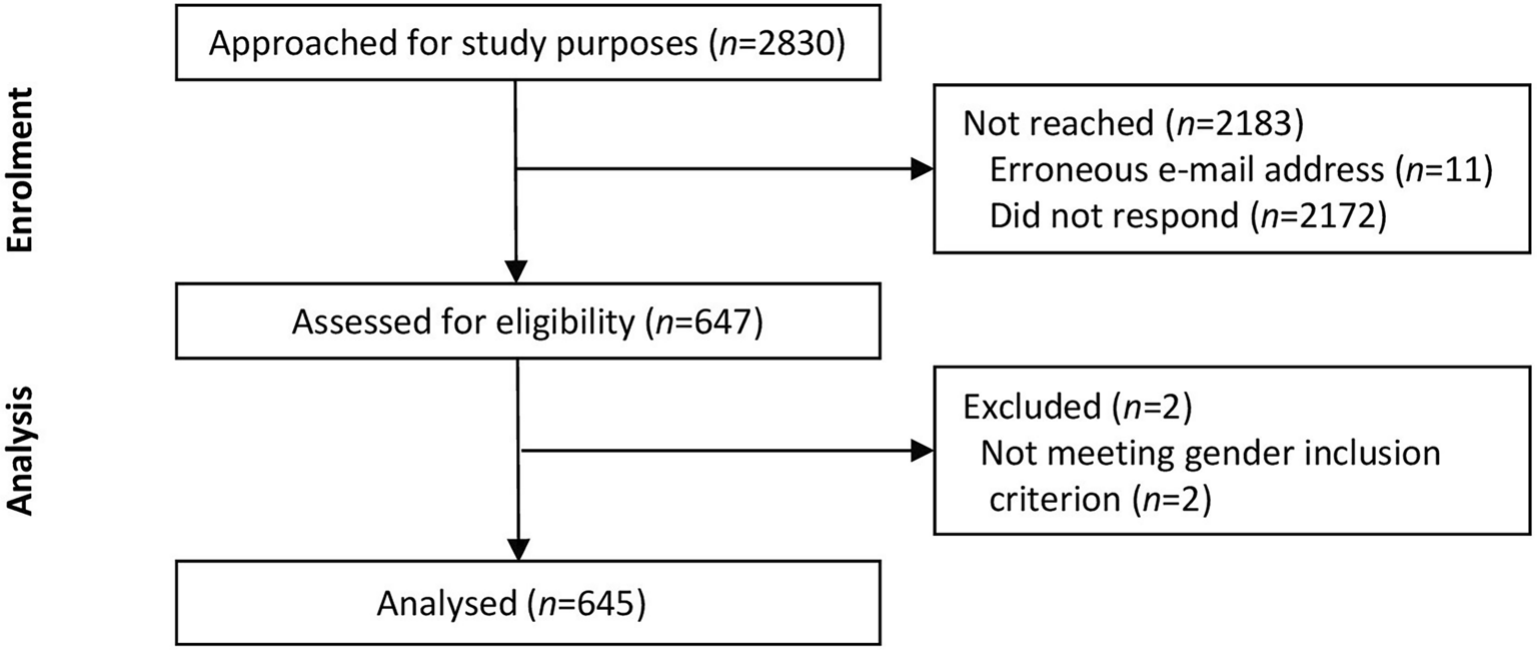
Recruitment flow chart.

The study was nested in the recruitment and eligibility phase of a randomized controlled trial on the effects of a psychosocial intervention on women’s well-being and ART outcomes (Clinical Trials.gov: NCT04151485). The study was approved by the Semmelweis University Regional and Institutional Committee of Science and Research Ethics, Budapest (reference number: 83/2019) and was carried out in accordance with the tenets of the Declaration of Helsinki. The research is reported in conformity with the COnsensus-based Standards for the selection of health status Measurement INstruments (COSMIN) reporting guideline for studies on measurement properties of patient reported outcome measures^36^. The inclusion criteria were as follows: (1) female sex; (2) reproductive age (18 to 45 years); (3) fluency in Hungarian; and (4) failure to achieve pregnancy after 12 or more months of regular unprotected sexual intercourse^37^. Women treated in the Assisted Reproduction Center of the Department of Obstetrics and Gynecology of Semmelweis University, Budapest, between December 2019 and February 2023 (*n* = 2830) were consecutively contacted via email directing patients to a web survey. Sample size was determined so as to fall in between those in previous validation studies of the SCREENIVF^38^. Eleven addresses were erroneous; thus, 2819 emails were successfully sent. Participation was voluntary and anonymous and based on informed consent after learning about the purpose and data management of the research. The overall response rate was 22.9%, resulting in a sample of *n* = 647 responders. Questionnaires were completed online, designed in a way that missing data were not allowed. Two men were found to have provided data, which were removed from analysis. The final sample consisted of *N* = 645 participants (Fig. 2).

### Measurement instruments

#### SCREENIVF

The 34-item questionnaire^16^ consists of five subscales measuring five risk factors: (1) *anxiety* (10 items originating from the Spielberger State-Trait Anxiety Scale^39^); (2) *depression* (a 7-item version of the Beck Depression Inventory for screening among primary care patients^24^); (3) *perceived social support* (5 items, derived from the Inventory of Social Involvement^40^); and infertility-related cognitions of (4) *helplessness* (6 items) and (5) *acceptance* (6 items), all from the Illness Cognition Questionnaire for IVF patients^41,42^. Each subscale preserves the original question and response format of its parent questionnaire. Cutoffs on the subscales differ from culture to culture. Dichotomous scores are given on each risk factor: 1 if the patient’s score falls in the critical range on the respective subscale, and 0 if not. Adding up all subscale scores yields the final Risk Factors score ranging between 0 (no risk factor) and 5 (5 risk factors). Subjects are considered to be at risk when scoring above 0. Cronbach’s alpha values in our sample ranged between 0.81 and 0.93.

#### Spielberger State-Trait Anxiety Inventory (STAI)

The Spielberger State-Trait Anxiety Inventory^39,43^ assesses anxiety on two 20-item scales: the State Scale (STAI-S), measuring transient states of subjective fear, tension and vegetative excitement, and the Trait Scale (STAI-T), capturing a more stable tendency of an individual to become anxious. All questions are answered on 4-point Likert scales. The results on both scales can range from 20 to 80, where a higher score indicates greater levels of anxiety. Form X, freely available and most widely administered in research and practice in Hungary, was used in this study. Cronbach’s alpha values in our sample were 0.95 and 0.92, respectively.

#### Beck Depression Inventory (BDI)

The Beck Depression Inventory^44,45^ contains 21 items with 4-point Likert scale responses about symptoms of depression, such as pessimism, lack of satisfaction, and guilt. Results can range from 0 to 63. Conventional cutoff scores on the BDI yield the following categories: normal range (0–9 points), mild (10–19 points), moderate (20–29 points), and severe depression (30–63 points). The nonclinical/clinical cutoff of 18/19 routinely applied in Hungarian studies^46^ is almost identical to the 19/20 cutoff suggested by Beck and associates^44^. In the present sample, the questionnaire yielded a Cronbach’s alpha score of 0.90.

#### Multidimensional Scale of Perceived Social Support (MSPSS)

The Multidimensional Scale of Perceived Social Support^47,48^ consists of 12 items across 3 subscales, assessing the individual’s subjective social support from family, friends, and significant others on 8-point Likert scales. The higher the mean on a factor or altogether, the stronger the support perceived by the responder. Cronbach’s alpha value in our sample was 0.87.

#### Fertility Quality of Life scale (FertiQoL)

The FertiQoL^14,17^ is a 36-item instrument for the assessment of the fertility-specific quality of life (QoL) of individuals. The tool contains 2 general items, a core and an optional treatment section. Core FertiQoL is composed of four subscales: Emotional; Mind–body; Relational; and Social. Treatment FertiQoL comprises two subscales: Environment and Treatment tolerability. Response formats follow 5-point Likert scales. All scale scores range between 0 and 100, with higher scores indicative of better QoL. In the present study, only the Core FertiQoL was used. Internal reliability was 0.90 for the module and ranged between 0.75 and 0.86 for the subscales.

Sociodemographic information such as age, marital status, number of existing children, residence, education, employment status, personal perception of financial situation, and health information such as height, weight, duration and etiology of infertility were gathered.

### Translation and adaptation

After obtaining permission via email from the developer of the original questionnaire, adaptation and translation were performed on version English 2.0 of the SCREENIVF following international recommendations^49,50^. Anxiety and depression subscale items were identified within the extant valid translations of the STAI and the BDI. For the remaining three subscales, the following steps were taken: forward translation by two translators fluent in both languages; consensus Hungarian version by a psychologist and a linguist; backward translation by two bilingual linguists; final version agreed upon by an expert committee of leading psychologists proficient in English and familiar with the research topic; and finally, pilot testing for comprehensibility, face validity and cultural appropriateness with 20 ART patients.

### Descriptive statistics

Statistical analyses were performed with IBM SPSS for Windows, v20.0^51^, and the lavaan R package^52^. All scale data were tested for normality of distribution with Shapiro‒Wilk tests.

### Reliability and validity

Internal consistency was measured with Cronbach’s alpha computed on the basis of the factor loadings of each item in a subscale. Reliability is acceptable when Cronbach’s alpha is higher than.70, and values > 0.80 are commonly considered good^53^.

To test concurrent validity, Spearman rank coefficients were computed to assess correlations between SCREENIVF subscales (not normally distributed) and their corresponding criterion measures. To avoid overlap, items used in the SCREENIVF were removed from the BDI and the STAI, and correlation tests were run with the bulk of remaining items only.

As for construct validity, since the SCREENIVF is a compilation of preexisting, unidimensional scales, and its underlying factor structure has already been identified in earlier studies, exploratory factor analysis is no longer necessary^54^. To verify the postulated underlying latent constructs in the Hungarian version, confirmatory factor analysis (CFA) was performed. Instead of the maximum likelihood (ML) method, which assumes that the observed indicators follow a continuous and multivariate normal distribution, diagonally weighted least squares (DWLS) was used, a method suggested to be superior to ML when ordinal data are analyzed^23^. All five factors were allowed to correlate. No cross-loadings or correlated errors were allowed. The goodness of fit of the model was evaluated by χ^2^ tests, the standardized root mean square residual (SRMR), root mean square error of approximation (RMSEA), and the comparative fit index (CFI). A model is considered to show good fit with SRMR/RMSEA < 0.08, and CFI/TLI > 0.90/0.95^55–57^.

### Cutoffs, sensitivity and specificity

A review of cutoffs applied in the original^16^ and the validation studies of the SCREENIVF^18,20–22^ was performed to inform thresholds for use in the present study. First, cutoff points for all Hungarian SCREENIVF subscales were calculated on the basis of sample means plus/minus standard deviations (M ± SD), as adequate. In the case of the Depression subscale, two other cut-points were also tested: 3/4, suggested by the developers of the SCREENIVF following Beck and colleagues^24^, and 4/5, a demarcation point resulting from a two-step cluster analysis that reveals natural groupings in a dataset. A similar cluster analysis was also performed on the SCREENIVF Risk Factors scale results.

Sensitivity and specificity measurements were performed for depression, the most commonly used indicator of psychological maladjustment in infertility, which, unlike anxiety, has internationally accepted scale cutoffs as reference points. Sensitivity here refers to the ability of the SCREENIVF to correctly identify patients falling into the ‘clinically depressed’ category on the BDI, while specificity refers to its ability to correctly identify patients without a mood disorder. Binary logistic regression was used to test the power of the SCREENIVF with different cut points to predict BDI depression categories. Receiver operating characteristic (ROC) curves were calculated for all SCREENIVF subscales and the Risk Factors scale, with BDI scores serving as a reference. ROC curves are a plot of false positives against true positives, where the closer the area under the curve (AUC) of a test is to 1.0, the better it is in terms of sensitivity and specificity^58^. AUC is heuristically interpreted as small (0.5 < AUC ≤ 0.7), moderate (0.7 < AUC ≤ 0.9), or high (0.9 < AUC ≤ 1)^59^. Youden Indexes of different cut-points were computed^60^. For all analyses, p < 0.05 was considered an indication of significance.

### Supplementary Information

Supplementary Information.

## Data Availability

The data that support the findings of this study are available and can be requested by academic researchers from the corresponding author.
