# Supplementary material for: The SCREENIVF Hungarian version is a valid and reliable measure accurately predicting possible depression in female infertility patients
Source: Sci Rep. 2024 Jun 5;14:12880. doi: 10.1038/s41598-024-63673-w (PMC11153651; doi:10.1038/s41598-024-63673-w)

The SCREENIVF Hungarian version is a valid and reliable measure accurately predicting possible depression in female infertility patients

Judit Szigeti F.*, PhD, PsyD, Institute of Behavioural Sciences, Semmelweis University, Üllői út 26, 1085 Budapest, Hungary; Department of Otorhinolaryngology, Head and Neck Surgery, Semmelweis University, Üllői út 26, 1085 Budapest, Hungary

Réka E. Sexty, PhD, Department of Psychology, University of Graz, Dachgeschoß - 2. Stock, 2, 8010 Graz, Austria; reka.sexty@uni-graz.at

Georgina Szabó, MA, MSc, Doctoral School of Mental Health Sciences, Semmelweis University, Üllői út 26, 1085 Budapest, Hungary; North Buda Saint John's Hospital Center and Outpatient Clinic, Department of Psychiatry, Diós árok 1-3, 1125 Budapest, Hungary; georginee@gmail.com

Csaba Kazinczi, MA, Department of Clinical Psychology, Semmelweis University, Budapest, Hungary; Doctoral School of Clinical Medicine, University of Szeged, 6722 Szeged, Hungary; kazinczicsaba.pszichologus@gmail.com

Zsuzsanna Kéki, MSc, Directorate for Human Reproduction, National Directorate General for Hospitals, Buda-part tér 2, BudaPart Gate Irodaház A. ép. 406, 1117 Budapest, Hungary; keki.zsuzsanna@okfo.gov.hu

Miklós Sipos, PhD, Assisted Reproduction Center, Department of Obstetrics and Gynecology, Semmelweis University, Üllői út 26, 1085 Budapest, Hungary; sipos.miklos.dr@gmail.com

Péter Przemyslaw Ujma, PhD, Institute of Behavioural Sciences, Semmelweis University, Üllői út 26, 1085 Budapest, Hungary; peteru88@gmail.com

György Purebl, PhD, Institute of Behavioural Sciences, Semmelweis University, Üllői út 26, 1085 Budapest, Hungary; purebl.gyorgy@gmail.com

Supplementary information

Table S1

Title: Item and item-subscale statistics for the Hungarian version of the SCREENIVF

SCREENIVF subscales and item content	M[a]	± SD[b]	Corrected item-subscale correlation	Cronbach's alpha if item deleted
**Anxiety (A[c])**				
A1 feeling fine	2.20	.936	.672	.875
A2 feeling satisfied	2.47	.963	.699	.873
A3 worrying too much	2.28	.968	.590	.880
A4 being happy	2.38	.956	.694	.873
A5 disturbing thoughts	2.32	.984	.621	.878
A6 feeling safe	2.04	.910	.509	.885
A7 feeling pleased	2.59	1.012	.653	.876
A8 haunting thoughts	1.83	.896	.450	.889
A9 taking disappointments seriously	2.04	1.033	.662	.875
A10 worried about current troubles	2.40	1.053	.690	.873
**Depression (D[d])**				
D1 sadness	.54	.608	.666	.766
D2 negative view of future	.72	.727	.585	.778
D3 failures	.61	.684	.574	.780
D4 anhedonia	.66	.748	.583	.779
D5 self-disappointment	.48	.761	.575	.781
D6 self-blame	.44	.677	.509	.792
D7 suicidal ideation	.09	.300	.381	.814
**Social support (S[e])**				
S1 someone to help one when tense	3.39	.832	.739	.886
S2 someone to talk to about nice experiences	3.79	.538	.656	.902
S3 someone to comfort one when in pain	3.47	.781	.825	.863
S4 someone to talk to when sad	3.52	.751	.854	.857
S5 someone to help one with difficult job	3.55	.724	.736	.883
**Helplessness cognitions (HC[f])**				
HC1 missing out on important things	2.20	1.016	.595	.857
HC2 fertility problem controlling life	2.14	.948	.682	.842
HC3 feeling useless	2.00	.014	.624	.852
HC4 life incomplete	2.65	.019	.711	.836
HC5 affect important aspects	2.13	.014	.743	.831
HC6 feeling helpless	2.64	.057	.634	.850
**Acceptance cognitions (AC[g])**				
AC1 ability to deal with consequences	2.22	.835	.685	.928
AC2 ability to live with fertility problem	2.07	.923	.795	.915
AC3 learning how to accept fertility problem	1.95	.871	.834	.910
AC4 ability to accept fertility problem	1.98	.933	.839	.909
AC5 cope with fertility problem even if not solved	2.09	.890	.771	.918
AC6 ability to cope with fertility problem	2.16	.847	.827	.911

Note. [a]Mean; [b]Standard deviation; [c]Anxiety subscale item; [d]Depression subscale item; [e]Social support subscale item; [f]Helplessness cognitions subscale item; [g]Acceptance cognitions subscale item.

Table S2

Title: Sensitivity and specificity statistics for different cutoff points on the SCREENIVF Depression subscale and Risk Factors scale

		**SCREENIVF Risk Factors Scale**		**SCREENIVF 'At risk' c/o[a] 0/1**			**SCREENIVF 'At risk' c/o 1/2**		
**SCREEN-IVF Depression subscale cutoff**	**BDI[b] Category**	**Predictive power (BLR[c])**	**AUC[d] in ROC[e]-analysis**	**Predictive power (BLR)**	**Youden -index**	**'At risk' population rate**	**Predictive power (BLR)**	**Youden -index**	**'At risk' population rate**
**3/4** (Beck et al., 1997)	C/NC[f] (c/o 9/10)	84.5 B=1.862 Wald= 158.99	.877 [CI[g]: .849-.905]	84.5 B=3.395 Wald= 242.70	J=69.0	50.9%	77.4 B=3.399 Wald= 139.57	J=54.7	31.9%
	NM/MS[h] (c/o 18/19)	86.7 B=1.376 Wald= 144.80	.905 [CI: .880-.930]	80.5 B=5.271 Wald= 27.342	J=60.1		81.4 B=3.100 Wald= 133.84	J=64.8	
**4/5** (Cluster analysis)	C/NC (c/o 9/10)	82.3 B=1.787 Wald= 139.95	.848 [CI: .817-.879]	82.3 B=3.155 Wald= 218.09	J=64.6	42.0%	76.0 B=3.557 Wald=118.61	J=51.9	29.3%
	NM/MS (c/o 18/19)	87.6 B=1.434 Wald= 151.28	.918 [CI: .895-.940]	80.5 B=5.592 Wald= 30.75	J=67.4		83.1 B=3.116 Wald= 144.61	J=65.1	
**6/7** (M[i]+SD[j])	C/NC (c/o 9/10)	77.8 B=1.636 Wald= 109.39	.795 [CI: .760-.831]	77.8 B=2.757 Wald= 170.46	J=55.6	39.2%	69.8 B=3.143 Wald= 85.24	J=39,4	22.8%
	NM/MS (c/o 18/19)	88.5 B=1.462 Wald= 155.54	.898 [CI: .863-.932]	80.5 B=3.363 Wald= 103.28	J=64.7		87.4 B=3.380 Wald= 174.41	J=66.4	

Note. All models were significant at the p<.01 level. The best sensitivity was reached with the SCREENIVF Depression subscale cutoff of 3/4 and the Risk Factors scale cutoff of 0/1. The best specificity was reached with the SCREENIVF Depression subscale cutoff of 6/7 and the Risk Factors scale cutoff of 1/2. [a]cut-off; [b]Beck Depression Inventory; [c]binary logistic regression; [d]area under curve; [e]receiver operating characteristic; [f]Case vs No-case; [g]confidence interval; [h]No-to-mild vs Moderate-to-severe; [i]Mean; [j]Standard deviation.

Figure S1

Title: Two-step cluster analysis of the SCREENIVF Risk Factors scale with a 6/7 (mean+ standard deviation) cutoff on the Depression subscale

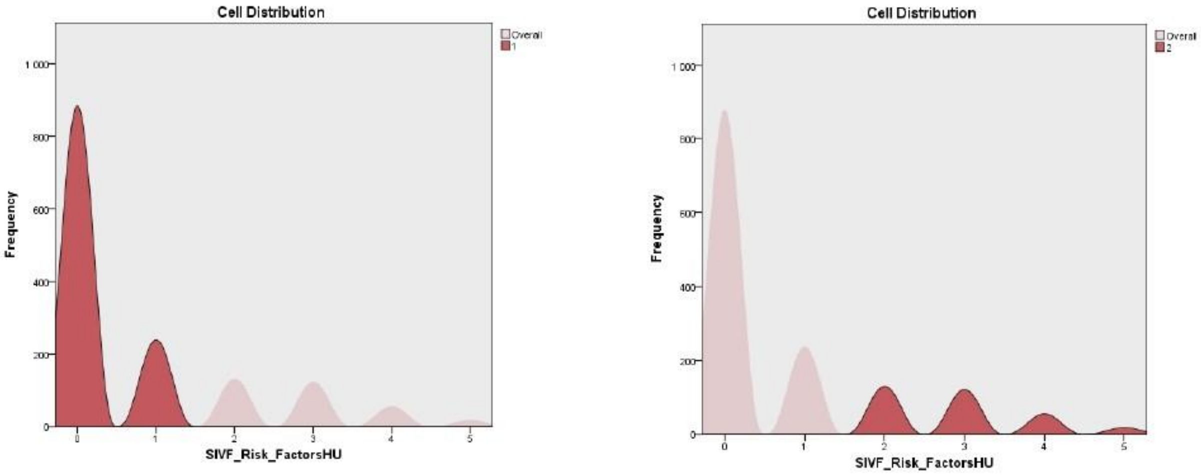

Figure S2

Title: ROC curves for total and individual SCREENIVF risk factors

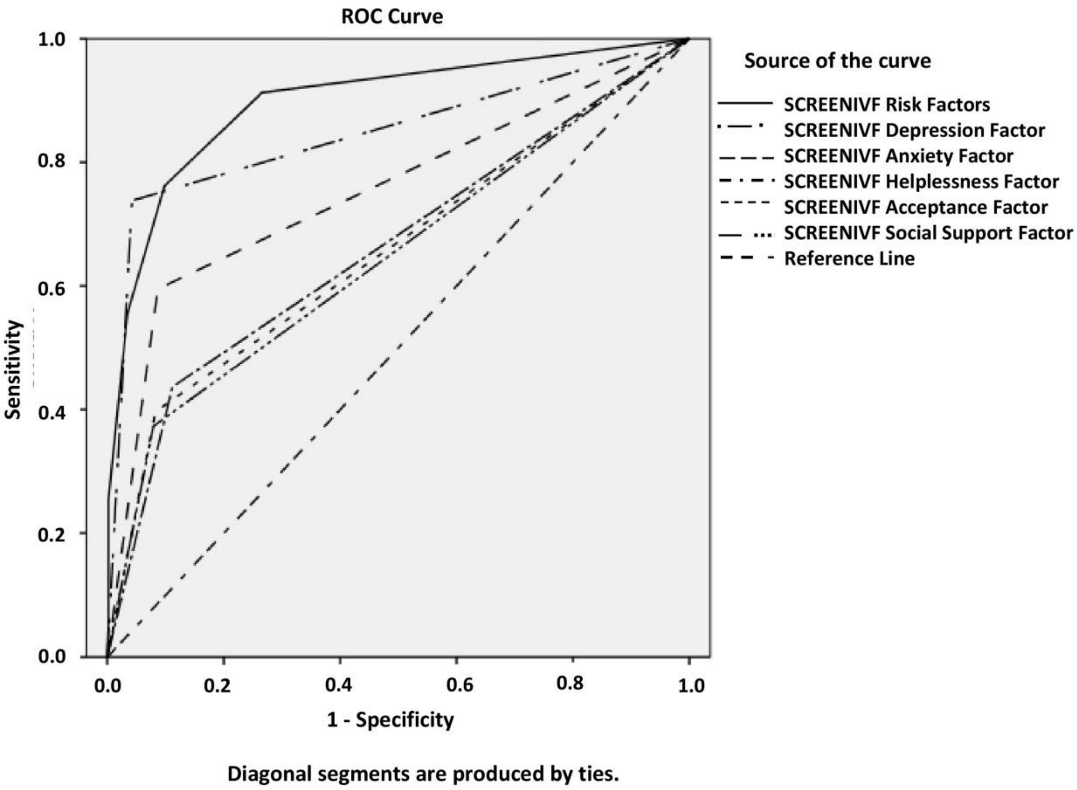

Supplement: Supplementary file 1 — Supplementary Information. [file 41598_2024_63673_MOESM1_ESM.pdf]
